# Neuromonitoring in Children with Traumatic Brain Injury

**DOI:** 10.1007/s12028-023-01779-1

**Published:** 2023-06-29

**Authors:** Shruti Agrawal, Francisco Abecasis, Ibrahim Jalloh

**Affiliations:** 1 Department of Paediatric Intensive Care, Cambridge University Hospitals National Health Service Foundation Trust, Level 3, Box 7, Addenbrookes Hospital Hills Road, Cambridge, UK; 2 University of Cambridge, Cambridge, UK; 3 Paediatric Intensive Care Unit, Centro Hospitalar Universitário Lisboa Norte, Lisbon, Portugal; 4 Department of Neurosurgery, Cambridge University Hospitals National Health Service Foundation Trust, Cambridge, UK

**Keywords:** Pediatric traumatic brain injury, Intracranial pressure, Cerebral autoregulation, Transcranial Doppler, Neuromonitoring, Brain chemistry, Cerebral oxygenation

## Abstract

Traumatic brain injury remains a major cause of mortality and morbidity in children across the world. Current management based on international guidelines focuses on a fixed therapeutic target of less than 20 mm Hg for managing intracranial pressure and 40–50 mm Hg for cerebral perfusion pressure across the pediatric age group. To improve outcome from this complex disease, it is essential to understand the pathophysiological mechanisms responsible for disease evolution by using different monitoring tools. In this narrative review, we discuss the neuromonitoring tools available for use to help guide management of severe traumatic brain injury in children and some of the techniques that can in future help with individualizing treatment targets based on advanced cerebral physiology monitoring.

## Introduction

Traumatic brain injury (TBI) remains one of the main causes of trauma-related mortality and morbidity in children worldwide [1]. Children with severe TBI (sTBI) are managed in the pediatric intensive care unit (PICU) with a combination of therapeutic interventions aimed at preserving neuronal structure and/or function. These are aimed at reducing the progression of primary brain injury as well as occurrence and impact of secondary insults to improve neurodevelopmental outcomes [2]. Various monitoring tools are required to define ongoing changes in systemic as well as cerebral physiology, ideally in real time, which are used as therapeutic targets for neuroprotection. Although we have robust tools for monitoring systemic physiology (such as invasive arterial pressure, end tidal carbon dioxide, oxygen saturations, cardiac output etc.), monitoring the brain is fraught with challenges [3]. Multiple complex pathophysiological cascades (excitotoxicity, disruption of blood–brain barrier, neuroinflammation, cytotoxic and vasogenic cerebral edema, ischemia, energy failure, and neuronal cell death [4–6]) are triggered by the initial brain injury, and no single tool is sufficient to assess the spectrum of these pathophysiological changes. It is imperative to interpret information available from these multiple monitoring tools simultaneously for effective clinical use, and there is potential to individualizing therapy titrated to evolving disease process in individual patients.

The mainstay of managing TBI in the PICU is based on intracranial pressure (ICP) monitoring, which also helps in deriving cerebral perfusion pressure (CPP) as per the established ICP- and CPP-directed therapies [2]. The evolution of advanced neuromonitoring tools can give greater insight into cerebral oxygenation, perfusion, electrophysiology, and metabolism [7]. There have also been advances in neuroimaging, which is beyond the scope of this review. We are going to focus on neuromonitoring in the acute phase of TBI, in which computed tomography imaging remains the cornerstone as a quick imaging tool to define intracranial pathology.

In this review, we elaborate on different neuromonitoring tools available and discuss their physiological principles and practical application as per available evidence for children with TBI in the PICU (Table 1). We also propose a treatment algorithm based on some of the advanced methods and discuss potential future targets.

**Table 1:** Neuromonitoring modalities

Monitoring tool	Monitored physiology	Advantages	Limitations
Automated pupillometry	Brainstem reflex (pupil size and reaction)	Noninvasive, objective measurements NPi can be useful in predicting rise in ICP and response to osmotic therapy	Further large-scale studies needed before routine use of NPi in clinical practice
ONSD	ICP	Noninvasive, point of care, predict raised ICP	Operator dependent Age-related heterogeneity in ONSD and optic nerve anatomy with variable results in pediatric studies
Near-Infrared spectroscopy	Regional cerebral oxygenation	Noninvasive CA indices	Poor correlation in presence of hematoma/bleeding Limited spatial resolution Interference from extracranial tissue
Transcranial Doppler	Cerebral blood flow mean velocity	Noninvasive CA indices and vasospasm Noninvasive ICP and CPP	Operator dependent Intermittent
cEEG	Seizures, Background Electrical activity	Noninvasive, continuous Quantitative and qualitative assessment Can help with prognosis, depth of encephalopathy	Resource intensive Sedation can affect interpretation
IP ICP	ICP	Continuous, easy to insert, lower complications	Invasive Can only be zeroed before insertion, and there is drift over 5–7 days, making measurements inaccurate Probe placement can affect ICP reading in focal lesions
Intraventricular ICP/EVD	ICP	Global value, CSF diversion if required In vivo calibration	Invasive Technically more difficult to insert than IP catheters (especially with effaced ventricles) Increased infection/bleeding as compared to IP catheters Intermittent readings
PbtO_2_	Regional brain tissue oxygen tension	Direct measurements of PbtO_2_ Continuous, real time Can help prevent ischemia with hyperventilation	Invasive Regional values and probe position affect values No normative data
Cerebral microdialysis	Cerebral metabolites: glucose, lactate/pyruvate	Direct measurement of extracellular brain fluid Assessment of delivery and use, metabolic stress and energy failure	Invasive, intermittent, resource intense Focal measurements No data in pediatric TBI outside research setting No normative data
Intracranial EEG	Seizures, background electrical activity Spreading depolarization	Continuous, better resolution than surface EEG, can detect spreading depolarizations	Invasive, limited pediatric data
Laser Doppler flowmetry	CBF mean velocity	Microcirculatory blood flow changes	Invasive, limited pediatric data
Thermal diffusion flowmetry	CBF	Continuous, direct measurement of CBF	Invasive, limited pediatric data

*CA* cerebral autoregulation, *CBF* cerebral blood flow, *cEEG* continuous electroencephalography, *CPP* cerebral perfusion pressure, *CSF* cerebro-spinal fluid , *EEG*, electroencephalography, *EVD* external ventricular drain, *ICP* intracranial pressure, *IP* intraparenchymal, *NPi* neurological pupil index, *ONSD* optic nerve sheath diameter, *PbtO*_*2*_ brain tissue oxygenation, *TBI* traumatic brain injury

## Basic Neuromonitoring

### Clinical Neurological Examination

The initial clinical neurological examination (CNE) after trauma is crucial in defining the severity of brain injury. It forms the basis of TBI classification and helps in predicting outcome. The most helpful components of the CNE in TBI are level of consciousness, focal deficits, and motor and pupillary response [8, 9]. Although crude, the presedation and postresuscitation Glasgow Coma Scale is used internationally to assess the severity of TBI [2]. Specifically, the motor score of ≤ 3 in the Glasgow Coma Scale is highly predictive of the outcome [10, 11]. Pupillary reactivity and size are important brainstem reflexes and are markers of ICP (except in patients with direct orbital trauma). Pupillary reflexes are relatively well preserved, with most sedative/analgesic drugs used in the PICU (except thiopentone) [12]. Hence, serial pupillary assessment is used in all children with TBI who are sedated/neurologically depressed or admitted for observation for mild to moderate TBI. Anisocoria is suggestive of rising ICP and impending uncal herniation, which prompts urgent ICP-lowering management and further diagnostic tests, such as neuroimaging, to identify the cause of rising ICP to guide further management [2]. Bilateral fixed dilated pupils, which do not return to normal with treatment, are associated with very high mortality. An infrared automatic pupillometer gives more accurate results than a manual clinical examination with detailed information about pupillary reaction, percentage change in pupil size, speed of reaction, and neurologic pupil index and holds promise in predicting neurological deterioration, rise in ICP, response to treatment, and prognosis [13, 14].

Ongoing serial CNE can give important information about new or evolving deficits. It is however difficult in children with depressed consciousness either due to TBI or sedation as a part of treatment to decrease ICP/cerebral metabolic demand. It is debatable whether a sedation hold at regular intervals is beneficial in patients with TBI because it may increase ICP, derange cerebral perfusion, and worsen metabolic stress [15]. There are recommendations for daily sedation holds for CNE in adult patients with TBI in specific situations in which it should not be used [16]. There is no similar consensus for pediatric TBI, which in part could be related to more frequent use of analgosedation drugs with higher context-sensitive half-lives (e.g., midazolam and morphine infusions rather than propofol and remifentanil). Another important consideration for serial CNE in children is the lack of validated objective scoring systems, which would ideally incorporate the child’s developmental stage, underlying neurodevelopmental problems and the level of sedation. Several new scores have been developed with these themes in mind for use in sedated patients, and they also take the patient’s developmental stage into account. These show promising early results while awaiting validation in larger studies [17, 18].

### Intracranial Pressure

Intracranial pressure is a sum total of pressure exerted by the brain, blood, and CSF (cerebro-spinal fluid) volumes. Any increase in volume of any of these three compartments is compensated by a decrease or shift in one or both of the other two compartments. However, inside a relatively fixed skull, there is a relatively small compensatory reserve to accommodate for changes in volume beyond which ICP can rise sharply. The rising ICP leads to compression of the brain parenchyma and ischemia due to compromised blood supply; if left untreated, rising ICP eventually leads to cerebral herniation and death [19]. In TBI, multiple mechanisms contribute to high ICP: bleeding as a direct consequence of trauma (hematoma, contusions), acute hydrocephalous, and cytotoxic and vasogenic edema. Hence, monitoring and managing ICP are considered the cornerstones of management of TBI.

Continuous ICP can be monitored by different invasive transducer placements, the most common being intraparenchymal probes and intraventricular catheters. Intraparenchymal probes are easier to insert but cannot be rezeroed once inserted. Intraventricular catheters, commonly known as external ventricular drains (EVDs), give a global ICP reading and offer the advantage of therapeutic CSF diversion if required but can be technically difficult to insert, especially with effaced ventricles, and carry a higher risk of infection and hemorrhage. They also need to be closed to give an accurate and continuous ICP reading unless there is a transducer probe on the outside of the ventricular catheter [20]. Because of the dual advantage of EVD (global ICP reading and CSF diversion if required), EVD is considered a gold standard for monitoring and managing ICP. This has been challenged by two large comparative effectiveness studies recently (one each in children and adults) that failed to show benefit of early EVD insertion and CSF diversion over intraparenchymal ICP measurements, with some suggestion of higher complication rates and worse outcomes with EVD [20–22]. This will help inform the next iteration of TBI management guidelines.

Several noninvasive ICP measurement techniques have been developed. However, they only give intermittent ICP values and require validation in larger studies before being used in routine clinical practice. The most common among these is the optic nerve sheath diameter, as measured by ultrasonography and derived values from Transcranial Doppler (TCD) recordings [23–25].

Despite sufficient evidence confirming an association of ICP > 20 mm Hg with poor outcome from TBI [26–31], the evidence to support benefit of ICP-guided management is not robust in either children or adults [29, 32–35]. There is further uncertainty whether the treatment should aim at reducing ICP or manipulating associated reduction in CPP to avoid cerebral ischemia. However, monitoring ICP certainly informs timing of further neuroimaging and/or neurophysiological monitoring to identify the cause, as well as treatment decisions of rising ICP. After considering the available literature, the international guidelines for managing sTBI in children make a level III recommendation for continuous invasive ICP monitoring to allow timely delivery and titration of treatment to reduce the secondary brain injury [2].

There is emerging evidence to suggest that the currently set ICP treatment threshold of 20 mm Hg may be too high, especially in younger age groups, and should be lower in an age-dependent manner, possibly individualized depending on the intracranial compliance [2]. The outcomes are not only related to a single ICP cutoff value of 20 mm Hg but also related to the dose and duration of ICP spikes in relation to the intracranial compliance [36], as well as the response to treatment [28]. Whether we can improve neurodevelopmental outcomes by maneuvering these insults is yet to be proven. There is also ongoing work to understand if intracranial compliance would affect the tolerance of a certain ICP value [37], which we will discuss further in the Advanced neuromonitoring section.

### Cerebral Perfusion Pressure

Intracranial pressure monitoring also facilitates CPP calculation in real time. Because CBF (cerebral blood flow) is difficult to measure clinically, mean arterial pressure (MAP) or CPP (CPP = MAP − ICP) is used as a surrogate. Maintaining adequate CPP is one of the two most important physiological targets in managing children with sTBI. The target CPP can be achieved by a decrease in ICP, increase in blood pressure, or both. The intention is to avoid hypoperfusion, which could lead to ischemic damage, while avoiding hyperperfusion, which could worsen cerebral edema and also cause side effects from maintaining very high blood pressure [38]. The international guidelines suggest targeting CPP above 40 mm Hg regardless of age based on the studies showing more episodes of CPP below 40 mm Hg in nonsurvivors [27, 39, 40]. The upper limit of 50 mm Hg is set with an acknowledgment that children at either end of the age range may need lower or higher CPP [2, 40, 41]. There is some suggestion that maintaining age-appropriate CPP may lead to improved outcomes with CPP cut points increasing with age (< 2 years = 45 mm Hg, 2 to < 8 years = 57 mm Hg, ≥ 8 years = 68 mm Hg) [42].

## Advanced Neuromonitoring

### Cerebral Autoregulation and Intracranial Compliance

The high nutrient and oxygen demands of the brain require steady CBF. In health, this is maintained by a complex interplay of mechanisms defined as cerebral autoregulation (CA), which maintains a steady cerebral perfusion irrespective of the changes in systemic blood pressure or CPP. Impaired CA is common after TBI, and this makes the brain more vulnerable to secondary insults. The fixed age-related CPP targets as advised by the current guidelines may not account for impaired CA and predispose the brain to cerebral ischemia or edema [43]. Fixed CPP targets are also inadequate across the entire developmental trajectory from birth till early adulthood because their limited autoregulatory capacity further predisposes them to overperfusion or underperfusion [44]. Improved understanding of impaired CA in an injured brain, particularly in younger age groups, has instigated research on individualizing treatment targets for management of cerebral perfusion [45].

The clinical measurement of CA is based on the concept that within the patient’s autoregulatory range, the intracranial blood vessel diameter will change with changes in systemic blood pressure to ensure constant or stable CBF. This means that with intact CA, the fluctuations in systemic blood pressure will not be reflected in the CBF. Clinically, CBF is difficult to measure, and hence a surrogate of CBF or cerebral blood volume (such as ICP, CPP, regional oximetry, cerebral flow velocities measured by TCD, etc.) is used [46]. Various static and dynamic CA indices have been developed by studying these changes in the intracranial compartment against either spontaneous or induced changes in systemic blood pressure [47]. Dynamic indices offer the advantage of continuous real-time measurement and do not require blood pressure challenges, such as static indices, which could be potentially harmful in an unstable patient.

**Fig. 1:**
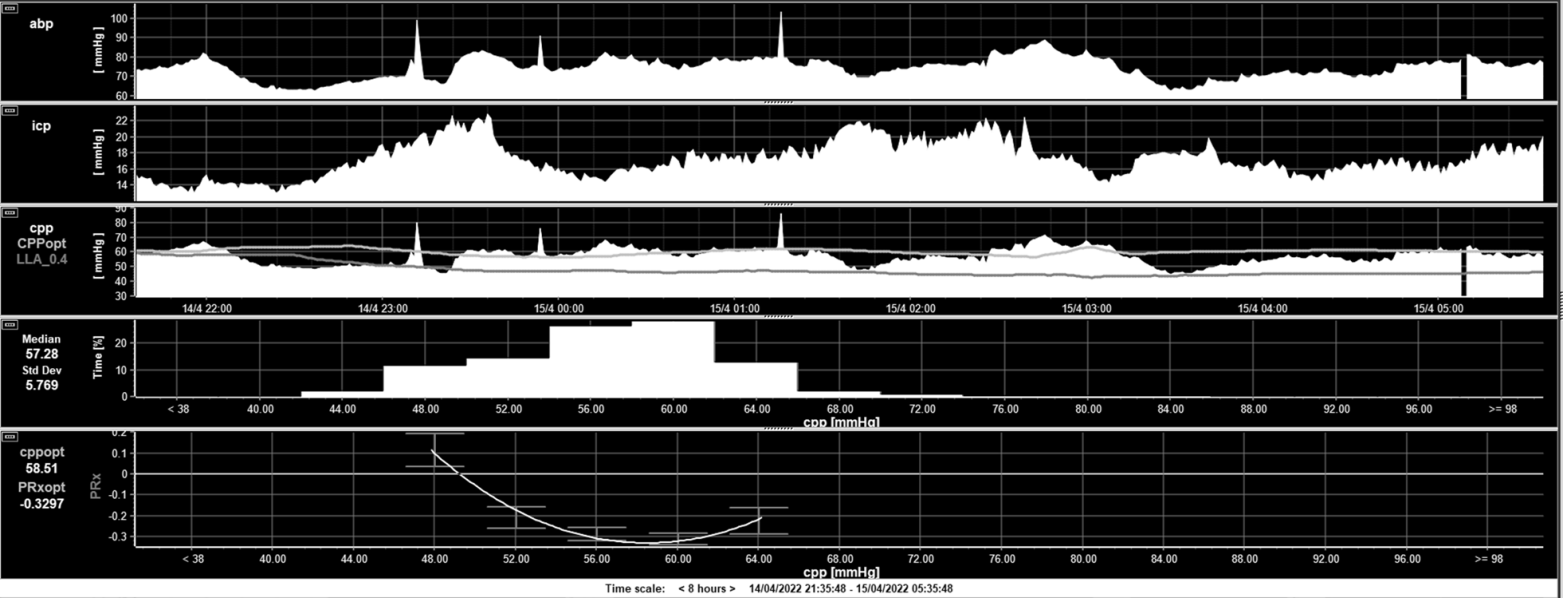
CPPopt calculation in real time in a child with sTBI. Real-time in-vivo calculation of CPPopt in a patient recruited to STARSHIP in an 8-h recording window. Top panel displays ABP, followed by ICP, and then CPP (CPPopt in the light gray line and lower limit of autoregulation in dark gray line). The bottom panel shows calculation of CPPopt. CPPopt is continuously calculated using a 5-min median CPP time trend alongside PRx. These PRx values are divided and averaged into CPP bins spanning 5 mm Hg. An automatic curve fitting method is applied to the binned data to determine the CPP value with the lowest associated PRx value. (Author’s own work). ICP intracranial pressure, CPP cerebral perfusion pressure, ABP arterial blood pressure, CPPopt optimum cerebral perfusion pressure, PRx pressure reactivity index, sTBI severe traumatic brain injury, STARSHIP Studying Trends of Auto-Regulation in Severe Head Injury in Paediatrics

Because all children with sTBI in the PICU have continuous ICP and ABP (arterial blood pressure) monitoring, these can be used to determine the dynamic state of CA. The most widely studied dynamic CA index is the cerebrovascular pressure reactivity index (PRx), which is a moving correlation coefficient between spontaneous slow wave changes in MAP and ICP over a 5-min window; this is updated every minute to give a continuous PRx reflecting the state of CA in real time [48]. This index can range from − 1 to + 1; negative or zero values indicate inverse relationship between ABP and ICP waveforms, signifying intact CA (associated with better clinical outcome), whereas positive values indicate transmission of changes in ABP waveforms to ICP waveforms, signifying disturbed CA (associated with poorer outcome) [49]. The PRx has shown an association with outcome in both adult and pediatric TBI [49–51]. Impaired CA does not directly affect the outcome, it is likely a reflection of increased vulnerability of an injured brain to secondary insults with an impaired CA. Although the PRx cannot be manipulated clinically, plotting measured CPP against the PRx yields a U-shaped curve, which can determine the lower and upper limits of autoregulation and CPP at which the vasculature is most reactive continuously in real time [52] (Fig. 1). This concept gives optimal CPP (CPPopt) for an individual patient at a particular point in time, which could be the optimum perfusion to the brain [53]. Interestingly, CPPopt has shown significant variation from the guidelines recommendation, and CPPopt can be different between patients and change over time in an individual patient. In addition, the difference between recommended CPP and CPPopt can be associated with worsened clinical outcome [50, 52]. For these reasons, indices of CA have been recommended to guide management of severe TBI in adults [54], but so far there is limited pediatric evidence to support its routine use in children.

A recent phase II study, COGiTATE (**C**PP**o**pt **G**u**i**ded **T**herapy: **A**ssessment of **T**arget **E**ffectiveness), confirms feasibility and safety of CPPopt-guided therapy in adult patients with TBI [54]. There are prospective multicenter studies currently underway to understand the relationship of CA-based indices in children with sTBI [55–57]; the PRx and CPPopt are being tested in children with sTBI in a prospective multicenter observational study, STARSHIP (**S**tudying **T**rends of **A**uto-**R**egulation in **S**evere **H**ead **I**njury in **P**aediatrics) in the United Kingdom, which will inform phase 2 and 3 studies in children [55]. The improved methods and understanding hold promise for future improvements in TBI management based on the state of intracranial physiology. This could be even more relevant in the pediatric population by potentially addressing the issue with fixed CPP targets across the developmental trajectory.

The model-based indices have evolved further by using the time domain and phase shift domain of the physiological waveforms. The newly described wavelet PRx is derived by phase shift between the two waveforms (ICP and MAP) and has shown promise in continuous calculation of CPPopt, with a better U-shaped fitting curve for establishing the CPPopt as well as upper and lower limits of autoregulation [58]. These indices have been used in a pediatric TBI cohort in a recent study with promising results [59]. There is evidence showing outcome association with ICP doses above the thresholds derived by graphing the PRx against ICP and likely reflects poor pressure tolerance in the state of impaired CA [60, 61]. The concepts and mathematical models that were thus developed have also been used to understand intracranial compliance RAP (correlation coefficient **R** between ICP **A**mplitude and mean IC**P**); intracranial compensatory reserve, RAC (correlation coefficient **R** between ICP **A**mplitude and **C**PP) [62]; and PAx (Pulse Amplitude Index which represents the relationship of ICP pulse amplitude with MAP) [63]. These studies hold promise for future use of individualized targets for ICP based on the state of intracranial compliance.

## Noninvasive Monitoring Methods

### Neurophysiology

Seizures are common in children after TBI [64]. They can be difficult to detect clinically because patients are deeply sedated as part of neuroprotective strategy. These subclinical seizures can increase ICP, worsen cerebral metabolic demands, and cause nonconvulsive status epilepticus. Hence, diagnosing and treating seizures are a part of treatment strategy for neuroprotection [2, 65]. Electroencephalography (EEG) is used to detect subclinical seizures as well as help with neuroprognostication from the background EEG patterns [66]. In monitored patients, as many as 30–40% patients with sTBI have been found to have subclinical seizures [67, 68]. EEG monitoring is also essential when barbiturate coma is used for refractory intracranial hypertension (ICH) to achieve burst suppression for maximum clinical benefit with the least side effects by using minimal dose. Continuous EEG is desirable but is resource intense and requires careful interpretation from experienced neurophysiologists [69]. Despite being limited to a few centers, accumulating experience in using continuous EEG has improved our understanding of degree of neurological dysfunction and background EEG patterns in brain injured children. In particular, background continuity and symmetry has been found useful in predicting outcome, with diffuse slowing, nonreactive pattern, severe burst suppression, and lack of sleep architecture associated with prolonged hospital stay, prolonged recovery, and poor outcomes [66, 70–72]. Easy accessibility and interpretation of amplitude-integrated EEG means it is increasingly used for monitoring children with sTBI. Additional information from quantitative EEG, such as power spectra within frequency bands, complexity measures, and suppression percentages, is useful to predict outcomes [73, 74]. Intracranial EEG can further improve seizure detection in comparison to surface EEG, although the experience in children is limited. This can also help detect cortical spreading depolarizations, which may not be detectable on surface EEG [75].

Evoked potentials can give useful information about brainstem function. Auditory and visual evoked potentials have been used to study the impact of mild TBI. Somatosensory evoked potentials have been used in predicting consciousness recovery and progression to a vegetative or minimal conscious state in sTBI, with normal somatosensory evoked potentials highly predictive of regaining consciousness [76, 77].

### Cerebral Oximetry

Near-infrared spectroscopy is a noninvasive technique of cerebral oximetry. It provides a global assessment of hemoglobin oxygenation in all vascular compartments (arterial, venous, and capillary) and can help assess cerebral hemodynamic and metabolic parameters [78]. The absolute cerebral oxygenation values have not proven useful from several studies in TBI, with disappointing results [79–83]. However, the trends and variation over time can aid clinicians in monitoring cerebral oxygen saturation and indirectly cerebral perfusion. It has also been proposed as a method to measure CA using the cerebral oximetry index, especially in situations in which invasive monitoring is not used [84, 85]. It has been studied in small pediatric TBI studies with promising results [41, 85–87], although larger pediatric studies are required before its clinical use.

### Transcranial Doppler Ultrasonography

Transcranial Doppler ultrasonography is used to calculate flow velocities in the cerebral arteries (cerebral blood flow velocity [CBFV]). Mainly the flow velocities of the middle cerebral and anterior cerebral arteries are measured to give an estimate of CBF [88]. It is used to calculate the pulsatility index (PI), which is the difference between systolic and diastolic middle cerebral artery flow velocity divided by mean flow velocity [88]. PI has been used to estimate ICP and CPP non invasively [88–91]. It is important to consider ABP and PaCO_2_ while calculating PI because they can give false negatives in cases of arterial hypertension and false positives in cases of hypotension or hyperventilation, respectively [91]. There is good accuracy of ICP estimated noninvasively by TCD ultrasonography in adult patients with TBI with a PI > 1.4 [92]. The results in children are however conflicting [90, 93, 94]. There is some suggestion that PI describes CPP more accurately than ICP in children, and several studies have tested the feasibility of TCD ultrasonography to estimate CPP [89, 93–95]. Indices of CA can also be calculated by using blood pressure and CBFV changes (autoregulation index) or signals of ABP and CBFV (Mx- Mean flow index) [96, 97].

Being a noninvasive technique, it can be extremely useful at admission to help determine the level of care and clinical priorities in children with TBI [98]. Insonating different arterial territories, it can help detect regional variations in cerebral hemodynamics and vasospasm.

### Invasive Monitoring (Brain Tissue Oxygenation and Cerebral Microdialysis)

Partial pressure of brain tissue oxygen (PbtO_2_) and cerebral microdialysis (CMD) provide real-time data on the cerebrovascular and metabolic status of brain tissue, respectively. These are increasingly recognized as the main pathophysiological processes, in addition to impaired perfusion from ICH, that cause secondary injury, with metabolic energy failure at the cellular level being the common end point for many of these processes. Directly monitoring tissue oxygenation and energy metabolism can potentially help individualize care rather than ICP monitoring alone. Both PbtO_2_ and CMD offer good temporal resolution but lack spatial resolution. The location of the monitors and their proximity to traumatic lesions need to be considered when interpreting the data. Although true normative values for PbtO_2_ and CMD are not known, increasing data on pathological thresholds has allowed development of treatment algorithms. Trends in the data over time are more informative than individual thresholds.

#### Brain Tissue Oxygenation

PbtO_2_ microsensors (such Integra Licox, Raumedic Neurovent) contain a miniature electrode that produces a small current proportional to the concentration of oxygen. Although regional, it provides valuable information about brain tissue hypoxia and mitochondrial failure, which can be used as a treatment target. It is also invaluable to prevent cerebral ischemia if hyperventilation is used as an ICP-lowering treatment [2].

In children, two cohorts provide most of the published data on PbtO_2_ monitoring. Stippler et al. did not find an independent association between tissue hypoxia and outcome in 46 children, but a PbtO_2_ of 30 mm Hg was associated with the highest sensitivity for favorable outcome at 6 months [26]. Interestingly, some patients with high ICP and reduced CPP (indicating compromised perfusion) had high rather than low PbtO_2_. Figaji et al. in 52 children demonstrated a significant relationship between PbtO_2_ and unfavorable outcome in a multivariate model (< 5 mm Hg for > 1 h or < 10 mm Hg for > 2 h) [40]. The latter study was confounded by the inclusion of PbtO_2_-targeted interventions in the treatment protocol. A later study of 81 children (including the 52 in the earlier study) from the same group found a nonlinear relationship between ICP and PbtO_2_ [99]. So the pediatric studies suggest a complex relationship and an association of PbtO_2_ with outcome [100]. This is reflected in the 2019 guidelines for the management of pediatric TBI, which support the use of PbtO_2_ in children (level III evidence) with a target of PbtO_2_ > 10 mm Hg (as suggested from the study by Figaji et al.) [2, 40]. The treatment threshold may need to be higher because PbtO_2_ values below 20 mm Hg are generally considered to indicate ischemia, reflected by published TBI treatment protocols that include PbtO_2_ (e.g., BOOST studies-**B**rain **O**xygenation **O**ptimisation in **S**evere **T**raumatic brain injury [101, 102]) or as measured by positron emission tomography imaging (ischemic threshold of 15 mm Hg or lower) [103].

#### Cerebral Microdialysis

Through continuous perfusion via a semipermeable membrane, the returning microdialysate from the CMD catheter can be analyzed at the bedside to measure several metabolites to reflect extracellular brain chemistry (glucose, lactate, pyruvate, glutamate, and glycerol). Glucose and the lactate/pyruvate (LP) ratio are most relevant and clinically useful. A low brain glucose level (neuroglycopenia) can be caused by a failure of perfusion (ischemia) or a low systemic (plasma) glucose level. The LP ratio is a marker of the cellular redox status, with an increased LP ratio reflecting either failure of oxygen delivery (ischemia) or failure of oxygen use (mitochondrial dysfunction). An LP ratio of more than 25 and a low brain glucose level of less than 0.8 mmol/L are considered pathological thresholds associated with unfavorable outcomes and necessitate intervention [104].

CMD catheters are implanted alongside ICP and PbtO_2_ sensors, often via a “bolt” with two to four lumens, and give hourly measurements. Recent developments can continuously analyze microdialysates using biosensors providing real-time data.

**Fig. 2:**
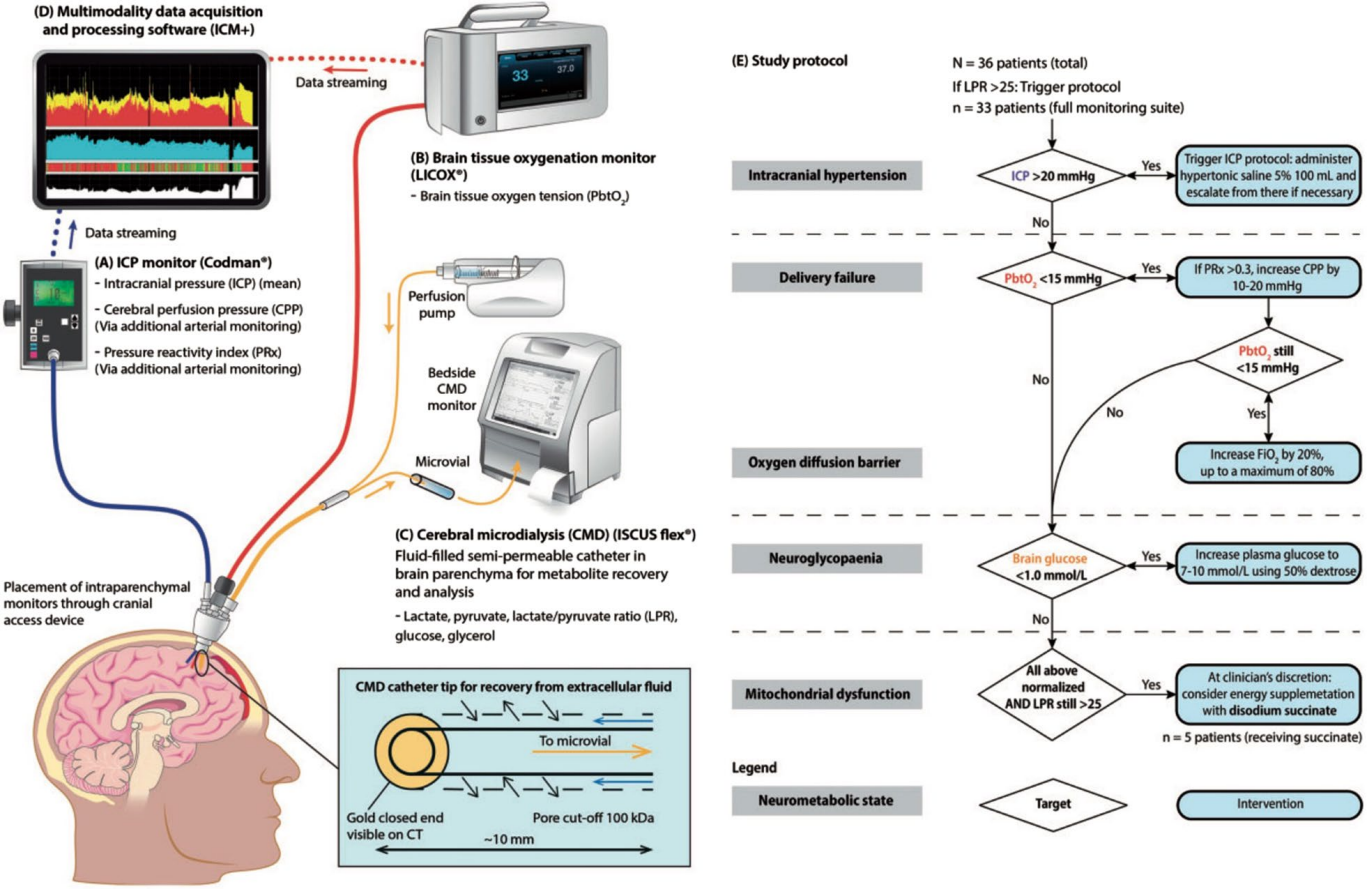
Clinical monitoring schema and protocol. Three intraparenchymal monitors are placed in the sedated, ventilated traumatic brain injury patient, via a cranial access device into the right frontal lobe. **a** Intracranial pressure is measured using a piezoelectric strain gauge (Codman). **b** Brain tissue oxygen is measured using a modified Clark electrode (Licox). **c** The cerebral microdialysis catheter (M Dialysis AB) consists of a double lumen catheter with a semipermeable membrane. A microfluidic pump perfuses the catheter with artificial brain extracellular fluid at 0.3 mL/h. The fluid recovered is collected in a microvial and assayed for lactate, pyruvate, glucose, and glycerol (bedside ISCUSflex analyzer). **d** Signals from intracranial pressure and brain tissue oxygen monitors are streamed in real time to a bedside computer with a multimodality data acquisition and processing software (ICM+) for analysis. **e** Study protocol for patients with raised LPR. Patients with cerebral LPR > 25 were treated in a staged fashion with the interventions within this flowchart. The neurometabolic state was classified in any given hourly time epoch, depending on the abnormalities defined above. CPP cerebral perfusion pressure; FiO_2_ fraction of inspired oxygen; ICP intracranial pressure; LPR lactate/pyruvate ratio; NMS neurometabolic state; PbtO_2_ brain tissue oxygen tension; PRx pressure reactivity index. Adapted from Khellaf et al. [110] under Creative Commons License (CC BY), published by SAGE, copyright the authors.

In large cohorts of adult patients, CMD, and in particular the LP ratio, has been robustly shown to independently predict outcome [105, 106]. Published CMD data in children are lacking, restricted to proof-of-concept and studies of specific metabolites of scientific interest rather than relevant to clinical management [107, 108]. The lower uptake of CMD compared to PbtO_2_ for clinical monitoring reflects, in part, the complexity of the pathophysiology that underlies a raised LP ratio and the lack of interventions that specifically target a raised LP ratio not caused by ischemia. A raised LP ratio in the context of adequate substrate delivery is termed mitochondrial dysfunction, an important component of metabolic failure in TBI. Interventions that target mitochondrial dysfunction are in development, necessitating more sophisticated protocols that incorporate CMD. An example of a treatment protocol that includes LP-ratio-directed therapy is provided in Fig. 2 [109].

## Future Directions

As discussed in this article, there are multiple monitoring techniques being used in clinical practice that give information about different pathophysiological pathways. A majority of these have limited value in isolation; it is important to interpret each of these in relation to other parameters being studied. As a baseline, children with sTBI have ICP monitoring, and we use an interlinked ICP and CPP targeted treatment algorithm as per the international guidelines [2]. There is an agreement that trends and waveforms give more useful information than the specific number, although bedside interpretation of the waveforms and trends can be difficult. New continuous monitoring software (ICM+, Moberg) offer the advantage of giving time trends, and the built-in software using machine learning can study different physiological waveforms in relation to one another. These have been developed to understand the state of CA and intracranial compliance as discussed in this article. They can offer valuable information about changing physiology in real time with an immense potential to individualize treatment targets for individual patients at specific points in time. Early results of use of these individualized targets are encouraging and will hopefully be incorporated in treatment algorithms in the near future to improve neurodevelopmental outcomes. The addition of other monitoring tools, such as PbtO_2_, TCD ultrasonography, EEG, CMD etc., requires complex treatment algorithms to help manage patients at the bedside. For example, if the ICP is high, additional information from TCD ultrasonography or PbtO_2_ can inform whether there is cerebral hyperemia (i.e., worsening cerebral swelling) or associated relative cerebral hypoperfusion, which will have a very different treatment strategy from the first scenario. This can also guide ICP lowering with hyperventilation while avoiding cerebral ischemia. One such treatment algorithm given by Appavu et al. elegantly incorporates additional information available from PbtO_2_, TCD ultrasonography/near-infrared spectroscopy, and EEG to manage raised ICP [110].

The evolution of both model-based indices and advanced monitoring techniques means there is a huge amount of data available at the bedside, some of it in real time, giving useful physiological information. Using this large amount of information for clinical decision-making still needs to be developed with the help of machine learning tools and then tested in clinical settings to prove benefit before mainstream clinical application.

A majority of recommendations for monitoring and management thresholds in the current international TBI guidelines from 2019 in children are level III because of the lack of high-quality studies [2]. As we have highlighted with individual neuromonitoring modalities, pediatric-specific evidence, especially through randomized control trials, is nearly nonexistent. To add to the complexity of doing research in children, the lack of equipoise in using individual neuromonitoring tools, which are becoming part of standard clinical practice in many institutes, makes it almost impossible to do interventional studies in TBI. This led to the largest multicenter international pediatric TBI study of comparative effectiveness, taking advantage of this heterogeneity and routine clinical use of different monitoring and management, ADAPT (**A**pproaches and **D**ecisions for **A**cute **P**ediatric **T**BI). The study recruited 1000 children with sTBI to help create level II recommendations for crucial therapeutic targets such as CSF diversion, hypoxia management, and ICH management [111]. The study has already reported on EVD vs. intraparenchymal ICP monitoring, which will help inform how we use these two tools [22]. Further results of this study will hopefully address some gaps in the literature.

## Conclusions

Children with sTBI require use of multiple systemic and neuromonitoring techniques. These give simultaneous information on different aspects of physiology, which can potentially affect patient outcomes. Advanced monitoring offers a distinct advantage by providing a direct assessment of the cerebrovascular and metabolic status of the brain. This is likely to be particularly important in children in whom fixed therapeutic targets may not address the developmental trajectory and changing physiology from birth to adulthood. The relationship between ICP, CPP, and outcome is not well defined and varies with age. A recent study demonstrated feasibility of implementing multimodal neuromonitoring in children with sTBI and, although a single-center study, shows promising results of utility in a clinical setting, with real-time physiology data informing bedside clinical decisions [66]. The evolution of multiple modalities to monitor the brain offers unique advantage in individualizing treatment. They hold promise for improving outcome pending accumulation of high-quality evidence confirming utility.
